# Infant Mortality Risk and Paternity Certainty Are Associated with Postnatal Maternal Behavior toward Adult Male Mountain Gorillas (*Gorilla beringei beringei*)

**DOI:** 10.1371/journal.pone.0147441

**Published:** 2016-02-10

**Authors:** Stacy Rosenbaum, Jean Paul Hirwa, Joan B. Silk, Linda Vigilant, Tara S. Stoinski

**Affiliations:** 1 Department of Anthropology, University of California Los Angeles, Los Angeles, California, United States of America; 2 Dian Fossey Gorilla Fund International, Musanze, Rwanda; 3 School of Human Evolution and Social Change, Arizona State University, Tempe, Arizona, United States of America; 4 Institute for Human Origins, Arizona State University, Tempe, Arizona, United States of America; 5 Department of Primatology, Max Planck Institute for Evolutionary Anthropology, Leipzig, Germany; 6 Dian Fossey Gorilla Fund International, Atlanta, Georgia, United States of America; University of Tasmania, AUSTRALIA

## Abstract

Sexually selected infanticide is an important source of infant mortality in many mammalian species. In species with long-term male-female associations, females may benefit from male protection against infanticidal outsiders. We tested whether mountain gorilla (*Gorilla beringei beringei*) mothers in single and multi-male groups monitored by the Dian Fossey Gorilla Fund’s Karisoke Research Center actively facilitated interactions between their infants and a potentially protective male. We also evaluated the criteria mothers in multi-male groups used to choose a preferred male social partner. In single male groups, where infanticide risk and paternity certainty are high, females with infants <1 year old spent more time near and affiliated more with males than females without young infants. In multi-male groups, where infanticide rates and paternity certainty are lower, mothers with new infants exhibited few behavioral changes toward males. The sole notable change was that females with young infants proportionally increased their time near males they previously spent little time near when compared to males they had previously preferred, perhaps to encourage paternity uncertainty and deter aggression. Rank was a much better predictor of females’ social partner choice than paternity. Older infants (2–3 years) in multi-male groups mirrored their mothers’ preferences for individual male social partners; 89% spent the most time in close proximity to the male their mother had spent the most time near when they were <1 year old. Observed discrepancies between female behavior in single and multi-male groups likely reflect different levels of postpartum intersexual conflict; in groups where paternity certainty and infanticide risk are both high, male-female interests align and females behave accordingly. This highlights the importance of considering individual and group-level variation when evaluating intersexual conflict across the reproductive cycle.

## Introduction

According to the sexually selected infanticide hypothesis, males kill infants when the infant’s death shortens the interval until the mother’s next conception, and the infanticidal male is likely to sire the mother’s next offspring [1]. In mammalian species in which the duration of lactation exceeds the duration of gestation, infanticide is common [2]. Infanticide accounts for a high proportion of infant mortality in a variety of species, including geladas (60%, [3]); solitary southern sea lions (23%, [4]); mountain gorillas (21%, [5]); lions (27%, [6]); chacma baboons; (31–76%, [7]); European rabbits (12%, [8]); wolverines (32%, [9]); white-faced capuchins (43–68%, [10]); and gibbons (83%, [11]). Sexually selected infanticide is believed to be a major selective pressure in primates, carnivores, and rodents (reviewed in [12, 13, 14]).

Female mammals use a variety of tactics to counter the threat of infanticide and reduce its impact on their fitness (reviewed in [15]). These strategies are deployed from the time of conception to weaning. In some species with promiscuous mating systems, females mate with multiple partners around the time of likely conception and during pregnancy in an apparent effort to confuse paternity (baboons: [16]; California ground squirrels: [17]; Japanese macaques: [18]; patas monkeys: [19]; bank voles: [20]; reviewed in [15, 21]). In other species, females terminate pregnancies when infanticide risk is high (voles & mice: [22, 23]; feral horses: [24]; gelada baboons: [25, 26]). After birth, females use maternal aggression (e.g. mice: [27]; ringtailed lemurs: [28]), coalitionary aggression (lions: [29, 30]; chimpanzees: [31]; blue monkeys: [32]), avoidance of potentially infanticidal males (alpine marmots: [33]; brown bears: [34]; ursine colobus: [35]), territoriality (white-footed and deer mice: [36]), and/or accelerated weaning (vervet monkeys: [37]; baboons: [38]) to reduce the risk to their infants.

Agent-based models and empirical evidence strongly support the hypothesis that lasting associations between males and females are an evolved anti-infanticide strategy in both monogamous and promiscuous species [11, 39, 40, 41, 42] (but see [43] for an alternative view). In monogamous systems, pair bonding across the entire reproductive cycle minimizes infanticide risk (e.g. [11]). In multi-male social systems, one important function of relationships between males and lactating females is thought to be defense against infanticide. Female chimpanzees [44, 45, 46, 47], lions [29, 48], gorillas [5, 40, 49], langurs [50] and baboons [7, 51] all rely on resident males to protect infants from infanticidal males.

In these promiscuous species, females may maintain close proximity to protective males to reduce the risk of infanticidal attacks by males who have recently immigrated into the group or gained high rank. For species in which females and infants can benefit from male protection, females may have incentive to encourage male behaviors that will protect offspring. In chacma baboons, close male-female associations, often called “friendships” [51, 52, 53], appear to exist primarily for infant protection. These relationships emerge after infants are born [7, 53] and are terminated after an infant dies [51]. Female baboons increase time spent with males after the birth of an infant, with the largest increase directed toward a single male partner; these males are often, but not always, the fathers of their female friends’ infants [54]. Once infants become partially independent, they continue to benefit from these protective relationships. Infants maintain closer proximity to their genetic father when their mother is out of view and another male is nearby than when no other males are in proximity [55].

Mountain gorillas (*Gorilla beringei beringei*) form single male or multi-male groups containing both related and unrelated adult males [56, 57] in which male-female bonds are stronger than same-sex bonds. In single male groups, all females have close relationships with the resident male. In multi-male groups, females typically spend more time in proximity to certain males than others [58, 59]. Most infants also spend more time near some adult males than others once they begin to move about independently [60, 61]. The primary function of such associations is thought to be infanticide avoidance (mountain gorillas: [40, 49, 62]; western lowland gorillas: [41]).

In the mountain gorilla groups monitored by the Dian Fossey Gorilla Fund’s Karisoke Research Center, infanticide is most likely to happen after a new male takes over a social group (usually due to the death of a dominant silverback or a group disintegration), or during an encounter between two social units [5]. Infants in single male groups are much more likely to die after a male replacement than infants in multi-male groups (57% of infants in single male groups die following replacements versus 6% in multi-male groups; [5]). During encounters between social units, mortality risk for infants is twice as high in single male groups as in multi-male groups (5.5% of infants versus 2.0%; [5]). Intragroup infanticide when a group male is the likely father is rare but has been reported [5].

For males, paternity certainty (and therefore incentive to protect infants) varies with group structure. Genetic paternity analyses confirm that all infants are sired by males in females’ social groups [63], so males in single male groups likely have very high paternity certainty. In multi-male groups, however, females mate promiscuously around times of probable conception [64, 65] and there is little mate guarding or mating harassment [64]. While dominant males sire the majority of infants [63, 66], reproductive skew can vary widely [67].

If infants benefit from association with males that can protect them against infanticide or other dangers (e.g. leopard predation; [68, 69], then mothers might attempt to encourage the development of these relationships in some way. Here, we test the hypothesis that females actively facilitate the development of relationships between infants and adult males by interacting selectively with certain males when their infants are young. We also evaluate the criteria mothers in multi-male groups use to select a preferred male partner when their infants are young. Finally, we evaluate whether maternal preferences in their infants’ first year of life are predictive of infants’ association patterns when they are older.

### Predictions

In both single and multi-male groups we expect females to spend more time near males when they had new infants than when they did not (replicating [58], but see also [59] and extending previous analyses to include additional behaviors and groups with more silverbacks). We also predict that females with new infants will spend more time actively affiliating (e.g. grooming, resting in contact) with male partners than other females do. When an infant is born into a multi-male group, a mother could encourage the development of an exclusive relationship with a specific male or encourage the development of relationships with several males. We predict that in multi-male groups, females will be more selective in their associations with adult males when they have a new infant than when they do not. Second, we expect that the male a mother spends the most time near during an infant’s first year of life will be the male her infant spends the most time near when they are 2–3 years old. At this age, infants are still nursing and potentially vulnerable to infanticide, but independently mobile.

Females are expected to prefer males who are most able and willing to provide protection for their infants. Gorilla mothers should display preferences for high-ranking males because they are likely to be in better physical condition than lower ranking males (e.g. ursine colobus: [35]; mountain gorillas: [5]). High-ranking males may also be more willing to incur the costs of defending infants because paternity is linked to male rank. Although mountain gorillas in this population had relatively high reproductive skew in the 1990’s [66], more recent work indicates that skew has fallen as both number of males in groups and male:female ratio has increased [63, 67]. However, it seems likely that rank remains an important cue for paternity. High ranking males still sire the majority of infants [63], and rank is the best predictor of male-infant relationship strength once infants are old enough to make independent social partner choices [67].

## Methods

This study was carried out in strict accordance with the laws of the countries in which it was conducted. The research protocol was approved by the University of California, Los Angeles Chancellor’s Animal Research Committee (protocol # 2011-003-01). Research was conducted on free-ranging animals in Volcanoes National Park, Rwanda, and was purely observational. Volcanoes National Park permissions were granted via letter from the Rwanda Development Board’s head of Tourism and Conservation.

### Subjects and data collection

This study was conducted on the habituated mountain gorilla population monitored by the Karisoke Research Center (KRC) in Volcanoes National Park, Rwanda. Data were collected by the first author (in 2003–04 and 2011–12) and second author (in 2011–12) via 50-minute focal animal follows on adult males and 2–3 year old infants. Additional focal animal data, including follows of adult females, were extracted from the KRC long term database, and include data collected between 2003 and 2013 by a variety of observers who passed repeated inter-observer reliability tests. All focal follows included 10-minute proximity point samples, in which the identity of all animals within 2m of the focal subject was recorded. Information about other forms of behavior, such as grooming duration, was not available from all of the long-term data, so analyses of these behaviors were limited to a subset of the complete dataset.

### Maternal categories

Mothers with infants 0–12 months old were considered mothers with young infants. Infant ages were known because KRC monitors individuals daily and maintains a long-term demographic database. Infants are much more reliant on their mothers in their first year than second. Before 6 months of age they virtually never move independently, but around 10 months of age they begin spending progressively more time off their mothers [70, 71]. At the same time infants begin spending more time away from their mothers, they also begin spending more time with adult males [60, 61]. Parous adult females with offspring older than 12 months, or without living offspring, are referred to as ‘other females’ or ‘females without young infants.’

Our sample contained 12 parous adult females living in single male groups, and 23 living in multi-male groups. All females contributed proximity data both when they were mothers of young infants and when they were not, but not all females contributed information about other behaviors in both conditions. Sample sizes are reported for each analysis.

Three females in multi-male groups were observed after the births of two different infants, and contributed two sets of paired data (with young infant, without young infant) to the dataset. We counted each of these as separate occurrences in our data summary. Thus, the total number of females in multi-male groups was 23, but the total number of data points was 26 for each female condition.

To control for group composition, we only used paired data from the same male-female dyad for time periods in which basic group composition was stable. For example, data were not compared before and after group fissions in cases where a dyad remained co-resident. Male-female dyads were excluded from the behavioral analyses if they were observed for less than 10 hours over a 6-month period in each maternal category (mothers of young infants: mean = 39.65 focal hours, min = 10.67, max = 85.67; other females: mean = 26.68 focal hours, min = 10.43, max = 74.52). For proximity analyses, dyads were excluded if there were not at least 100 point samples in each maternal category (mothers of young infants: mean = 385 point samples, min = 103, max = 1758; other females: mean = 373, min = 109, max = 989).

### Male categories

Adult males (hereafter also referred to as silverbacks) were included if they were at least 12 years old at the time of observation (n = 19 silverbacks, ages obtained from the long-term KRC demographic database). This followed age/sex classifications outlined in [72]. Although 12-year old males have not reached their maximum size [73, 74], they can copulate with adult females and sire offspring [63, 67], and take an active role in intergroup interactions, a situation in which infanticide risk is particularly high [49, 75, 76, 77].

### Group structure

Our analyses included four multi-male groups (mean = 4.25±0.83 males/group, 6.0±2.0 females/group), two single male groups (one containing two females, the other five), and one two-male group (containing 5 females) in which the beta male was very peripheral and very rarely interacted with other group members. This silverback was not included in the analyses reported below, and the group in which he lived was categorized as single male.

### Behavioral measures

We evaluated five behavioral measures to assess relationships between silverbacks and adult females: proportion of point samples in close (<2m) proximity, approaches and leaves, time spent resting in physical contact, time spent grooming, and following (see Table 1 for definitions).

**Table 1:** Behavior variable definitions.

Behavior	Definition	Directional?	Method
**Close proximity**	Within 2m of another animal, regardless of activity.	No	IPS*
**Approach-leave**	One animal moves within 2m of another animal and remains there at least 5 sec; one animal moves outside of 2m range after remaining there at least 5 sec.	Yes	CFS^
**Groom**	One animal manipulates another’s pelage with mouth or fingers.	Yes	CFS
**Rest in contact**	One animal rests any body part on any part of another’s body for at least 5 sec.	When observed	CFS
**Follow**	Individual gets up and walks directly in the path of an individual leaving a resting or feeding space. Must be within 2m and 5 sec of the first animal leaving; must walk in path for at least 5m.	Yes	CFS

*IPS = Instantaneous point sampling

^CFS = Continuous focal animal sampling.

### Genetic paternity determination

Fecal samples were collected from all infants, mothers and potential fathers for noninvasive genetic paternity analysis. Samples were preserved using the two-step ethanol-silica storage method described in [78]. We extracted DNA and genotyped samples at 16 autosomal microsatellite loci using the approach detailed in [79], including the appropriate amount of replication of results to avoid errors such as allelic dropout. Sex was determined or confirmed using a PCR-based sexing assay [80].

Individual IDs of samples were confirmed by comparing genotypes of known mother-infant pairs or by comparing the genotypes obtained from two or more samples purported to be from the same individuals. We considered all males 7 years of age or older resident in the group at the time of conception as potential sires [63]. There were one to 14 potential sires per offspring (mean = 5.8). We conducted likelihood assessment of paternity using CERVUS 3.0.3 [81]. We then conducted simulations assuming either 5 or 9 potential sires and assuming 10% of potential sires were related at the level of half-siblings (R = 0.25). The simulations assuming 5 and 9 potential sires were applied to datasets consisting of offspring with 6 or fewer potential sires and 7 or more potential sires per offspring, respectively. Results did not differ when we used simulations with different numbers of potential sires or increased proportions of relatives among the potential sires. We required assigned sires to be identified with 95% confidence by CERVUS. In addition to employing CERVUS to assess the statistical confidence of the paternity assignments, we compared offspring, mother and potential sire genotypes for genotypic incompatibilities (‘mismatches’).

Two of the 18 infants for which we were able to determine paternity were sired by males from outside the social groups they resided in. In both cases, their mothers had moved to the current group early in the pregnancy.

### Data analysis

Behavior data were summarized separately for each male-female dyad in each maternal condition. For one analysis in multi-male groups, we summed the proportion of time females spent in proximity to all available silverbacks in each maternal condition.

#### Behavioral measures

Not all individuals were observed for the same amount of time, so behavioral measures were adjusted to account for this variation. The proportion of samples in which male-female pairs were in close proximity was computed by dividing the number of point samples the animals were observed in close (2m) proximity by the sum of the number of point samples collected on each dyad partner (i.e. A&B together/(A observed + B observed). We also summarized proximity point sample data in the same manner for each male-infant dyad when the infant was 2 to 3 years old, to determine whether female preference predicted infant preference for individual males. The percentage of time spent grooming was calculated by dividing the duration of time the male-female dyad spent grooming by the sum of the duration of time the male was observed plus the duration of time the female was observed. The same procedure was used to compute the percentage of time spent resting in physical contact. Rates of approaches, leaves, and following were calculated by dividing the number of times the behavior was observed by the amount of time the male was observed plus the amount of time the female was observed. Only dyads that had ≥ 15 approach-leave interactions were included in approach-leave analyses.

#### Partner diversity

To determine whether the presence of young infants influenced females’ social partner diversity in multi-male groups, we calculated Shannon Weiner diversity index values (*H*) for each female in multi-male groups [82]:

$$H=\text{-}\sum \left[(p_{i})\times \ln\left(p_{i}\right)\right]$$

Where p_*i*_ was the amount of time a female spent in close proximity to silverback *i*, divided by the total time spent in close proximity to all available silverbacks. We used *H* to calculate an equitability score *E* [83]:

$$E=H/H_{max}$$

where *H*_*max*_ was the number of silverback social partners available to a female. Values closer to zero indicate that females distributed their time less equally across silverbacks; values closer to one indicate they distributed their time more equally. *E* scores were computed separately for mothers of young infants and other females.

#### Female choice rank

To determine whether females in multi-male groups were more likely to increase the proportion of time spent near certain preferred males when they had young infants, we calculated the percent change in the proportion of point samples these females spent in close proximity to each available male, relative to when they did not have young infants. We ranked all silverbacks available to each female by the proportion of point samples spent in close proximity to him when the female did not have a young infant (i.e. the silverback a female was most often in close proximity to received a 1, the silverback she spent the second-most time in close proximity to received a 2, etc.). If a female was equally likely to be in close proximity to two silverbacks, they both received the same rank. We grouped silverbacks by these “female choice” rankings. We then calculated the mean percent change in proportion of point samples between female conditions (with or without young infant) across all dyads that held the same female choice rank, to determine whether choice ranks were associated with changes in proximity.

#### Silverback dominance rank

For some analyses, silverbacks were grouped by dominance rank. Following methods used in many other publications [56, 57] dominance ranks were determined using non-aggressive displacement patterns. Displacement interactions typically follow an age-graded pattern, with older males dominant over younger ones [84, 85]. Males were categorized as alpha (rank 1, referred to here as dominant or α), beta (2 or β), gamma (3 or γ), and subordinate (>3 or δ). Due to the low number of displacements between some pairs of males it was impossible to accurately assign rank below gamma, so we grouped all males below the third-ranked position as ‘subordinate.’ [64]. During the study period a dominance change occurred between a dominant and a second-ranked silverback in a group that contained five males. Research staff from the Dian Fossey Gorilla Fund International used an Elo rating procedure [86] to determine the most likely date of the dominance change; the date calculated was the date used here [D. Caillaud, pers. comm.].

### Statistical analysis

It was clear from visual inspection of the data that patterns for dyads containing dominant silverbacks were quite different than patterns for dyads with other silverbacks. We separated the two categories of silverbacks for most analyses and present them separately unless otherwise stated.

The same basic structure was used for models across response variables. To determine if female condition (coded as a categorical variable where 0 = females without young infants, 1 = females with young infants), group type (categorical where 0 = single male groups, 1 = multimale groups), male dominance rank (coded as an ordinal variable where 0 = dominant, 1 = beta, 2 = gamma, 3 = subordinate), and/or female choice rank (ordinal where 0 = most preferred male) were related to our behavioral measures, we used one or more of these four predictor variables as fixed effects in the relevant multilevel mixed effects regression model(s). In most cases the dyad was the unit of analysis, so both partners’ identities were included as random effects to control for the repetition of individuals across dyads. Exceptions are described below.

All models were first estimated using an unrestricted means approach, where only the response variable and the random effects were included. Predictor variables were then added to address the hypotheses under consideration. Level one residuals were visually inspected with no major abnormalities found. There were some moderate outliers, but there was insufficient evidence to justify the removal of any data points. For all models containing at least one statistical trend or statistically significant predictor variable, we report the Wald test statistic and associated p value.

For data based on proportions (proximity data), we used logistic regression models. For data based on counts (following, approaching, and leaving behavior), we used Poisson regression models. For data based on percentages (resting in contact and grooming, change in proportion of point samples in close proximity) we used standard linear models. For these, since there was no temporal ordering of the observations the only possible residual structure was exchangeable or independent. Due to the complex nesting and unbalanced nature of the data, models with an exchangeable residual error would not converge. The independent residual error structure was thus adopted for all of the linear mixed models.

In one analysis, for each condition we summed the proportion of point samples females spent with all available male partners in multi-male groups. To evaluate whether the total (summed) proportions were different when females did and did not have young infants, we used a linear probability model. The summed probabilities for each female were well below 1 (range: 0.05–0.42). We included female identification as a random effect, since three females had data from more than one point in time. For the analysis comparing females’ equitability scores, we used a paired t-test.

For models evaluating dyads including dominant males, we included an interaction term for female condition and group type; for models testing dyads including non-dominant males, we included an interaction term for female condition and male rank. Models that included only dyads containing dominant silverbacks excluded rank (because all males hold rank 1); models that included only dyads containing non-dominant silverbacks excluded group type (because they only occur in multi-male groups).

To determine what criteria females used to choose their silverback social partners, we calculated adjusted Akaike information criteria (AICc) scores for multilevel mixed effects logistic regression models, where the outcome variable was proportion of point samples in close proximity. We evaluated models containing the predictors silverback dominance rank and genetic paternity individually, both variables together, and an interaction between the two. These were ordered according to AICc score. We then calculated the difference between AICc scores (ΔAICc); values of ≤2 indicate similar support for the corresponding models, with increasingly larger numbers indicating less support [87, 88]. We also calculated AICc weights (ωAICc), which can be interpreted as the likelihood that a given model is the best model of the candidates tested [87]. Finally, we used model averaging to determine the relative importance of our individual predictors, by calculating weighted mean estimates and standard errors for each parameter [89]. All analyses were performed using Stata 13 [90].

## Results

### Prediction 1

In all groups, females will spend more time near males when they have young infants than when they do not.

#### Dominant silverbacks

**Fig 1:**
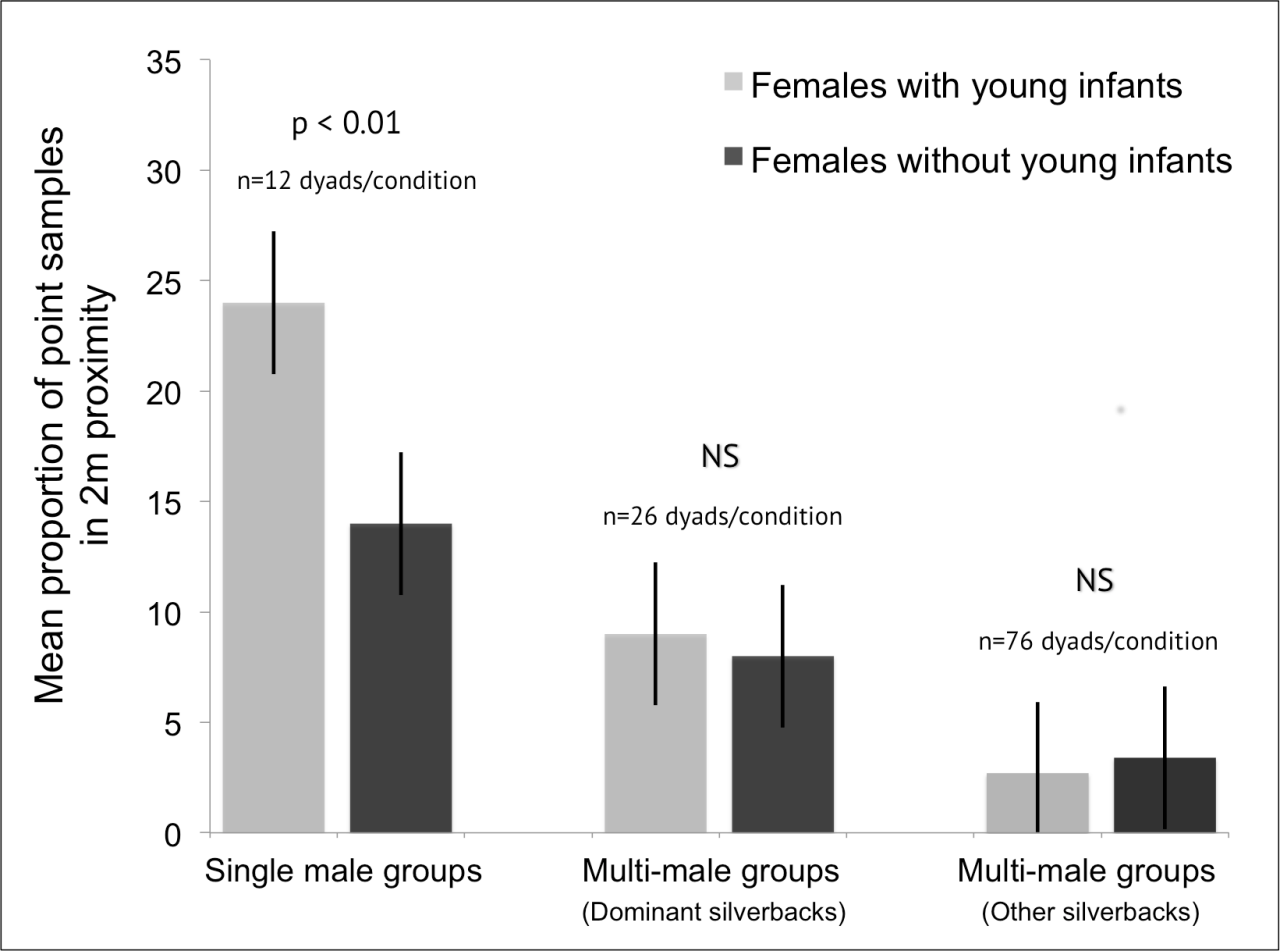
Females in single male groups were more likely to be in close (2m) proximity to males when they had a young infant. Females in multi-male groups were more likely to be near dominant males than non-dominant males, but the proportion of point samples they were near males was similar regardless of whether they had young infants (p = 0.100).

Proportion of point samples in close proximity: Across group types, females with young infants were more likely to be in close proximity to silverbacks than were females without young infants (Table 2A). However, females in single male groups drove the result; they were more likely to be in close proximity to dominant silverbacks than females in multi-male groups across both conditions (Table 2A, Fig 1). Females in single male groups were in close proximity to silverbacks more often when they had young infants (mean = 0.24% of point samples, SD = 0.05) than when they did not (mean = 0.14, SD = 0.08, n = 12 dyads per condition). In contrast, the presence of young infants had no consistent effect on females’ proximity to dominant silverbacks in multi-male groups (females with young infants: mean = 0.09, SD = 0.06; without young infants: mean = 0.08, SD = 0.05, n = 26 dyads per condition).

**Table 2:** Male-female dyads containing dominant silverbacks.

**2A** Proportion of point samples in 2m proximity (Wald chi2: 240.94, p<0.000)							
	**Coef**	**Std Err**	**Z**	**P>|z|**	**95% CI (lower)**	**95% CI (upper)**	**n (dyads)**
Female condition^t^	0.685	0.050	13.79	**0.000**	0.588	0.783	MYI^y^ = 38 WYI^ = 38
Group type^x^	-0.672	0.174	-3.87	**0.000**	-1.012	-0.331	Single male = 24 Multi-male = 52
Fem cond x group type	-0.522	0.069	-7.51	**0.000**	-0.658	-0.386	
Constant	-1.854	0.140	-13.24	0.000	-2.129	-1.580	
**2B** Rate of female approaches to dominant males (Wald chi2: 16.45, p = 0.003)							
Female condition	0.431	0.134	3.23	**0.001**	0.169	0.693	MYI = 15 WYI = 27
Group type	-0.551	0.177	-3.11	**0.002**	-0.898	-0.204	Single male = 15 Multi-male = 27
Constant	-1.513	0.130	-11.65	0.000	-1.767	-1.258	
**2C** Rate of females leaving dominant males (Wald chi2: 8.20, p = 0.017)							
Female condition	0.199	0.140	1.42	0.156	-0.076	0.473	MYI = 15 WYI = 27
Group type	-0.447	0.162	-2.76	**0.006**	-0.765	-0.130	Single male = 15 Multi-male = 27
Constant	-1.562	0.117	-13.37	0.000	-1.791	-1.333	

^t^ = Comparison of females with and without young infants; reference category was females without young infants.

^x^ = Comparison of single male versus multi-male groups; reference category was single male groups.

^y^MYI = Mothers of young infants.

^WYI = Females without young infants.

Rate of proximity initiation: Across group types, females with young infants approached dominant silverbacks at significantly higher rates (mean = 0.27/hour, SD = 0.15, n = 15 dyads) than did females without young infants (mean = 0.18/hour, SD = 0.11, n = 27 dyads) (Table 2B). Females in single male groups approached dominant silverbacks at significantly higher rates than did females in multi-male groups regardless of whether they had a young infant (single male groups: mean = 0.28/hour, SD = 0.14, n = 15 dyads; multi-male groups: mean = 0.18/hour, SD = 0.12, n = 27 dyads) (Table 2B).

There were insufficient females with young infants in single male groups with approach rate data available to test for a statistical interaction effect between female condition and group type. Within single male groups, females with young infants approached silverbacks a mean of 0.46 times/hour (SD = 0.10, n = 3 dyads). For females without young infants in these groups, the mean approach rate was half that, 0.23/hour (SD = 0.10, n = 12 dyads). Within multi-male groups, females with young infants also approached dominant silverbacks more often (mean = 0.22/hour, SD = 0.12, n = 12 dyads) than other females did (mean = 0.14, SD = 0.11, n = 15 dyads), but the difference between the two conditions was smaller.

Rate of proximity termination: Across group types, females with young infants left dominant silverbacks at the same rate females without infants did (with young infants: mean = 0.19/hour, SD = 0.10, n = 15 dyads; without young infants: mean = 0.17/hour, SD = 0.10, n = 27 dyads; Table 2C). Across female conditions, females in single male groups left dominant silverbacks at higher rates than females in multi-male groups did (single male groups: mean = 0.22/hour, SD = 0.11, n = 15 dyads; multi-male groups: mean = 0.15/hour, SD = 0.09, n = 27 dyads; Table 2C).

There were also insufficient dyads from single male groups containing females with young infants that had leaving rate data to test for a statistical interaction. In single male groups females with and without young infants did not appear to leave dominant silverbacks at different rates (with young infants: mean = 0.30, SD = 0.11, n = 3 dyads; without young infants: mean = 0.21/hour, SD = 0.10, n = 12 dyads). The same was true in multi-male groups; females with and without young infants left dominant silverbacks at similar rates (with young infants: mean = 0.17/hour, SD = 0.09, n = 12 dyads; without young infants: mean = 0.14/hour, SD = 0.09, n = 15 dyads).

#### Non-dominant silverbacks

Proportion of point samples in close proximity: Females were more likely to be in close proximity to higher-ranking non-dominant silverbacks than lower ranking ones (S1A Table). There was no difference in the proportion of point samples females were near all ranks of non-dominant silverbacks when they had young infants and when they did not (with young infants: mean = 0.03, SD = 0.05; without young infants: mean = 0.03, SD = 0.04, n = 76 dyads per condition). There was also no interaction effect between female condition and male rank (S1A Table, Fig 1).

Rate of proximity initiation: Rates of female approaches to non-dominant silverbacks were generally low (mean = 0.11/hour, SD = 0.13, n = 50 dyads). There was no relationship between non-dominant males’ rank and the rate at which females approached them (S1B Table). Females with young infants did not approach non-dominant males at different rates than females without young infants did (with young infants: 0.12/hour, SD = 0.14, n = 23 dyads; without young infants: 0.09/hour, SD = 0.12, n = 27 dyads; S1B Table). There was no interaction effect between female condition and non-dominant males’ rank (S1B Table).

Rate of proximity termination: The rate at which females left non-dominant silverbacks was similarly very low (mean = 0.12, SD = 0.14, n = 50 dyads), and females left non-dominant males at similar rates regardless of male dominance rank (S1C Table). Females with and without young infants left non-dominant males at the same rate (with young infants: mean = 0.14/hour, SD = 0.15, n = 23 dyads; without young infants: mean = 0.10/hour, SD = 0.14, n = 27 dyads; S1C Table). There was no interaction effect between male rank and female condition (S1C Table).

Since we found no difference in the proportion of point samples females with and without new infants spent in close proximity to either type of silverback (i.e. dominant or non-dominant) in multi-male groups, we also checked to see whether the proportion of point samples they spent near all males together changed. When the proportions for each female are summed across male partners, there was no difference in the proportion of point samples females were in close proximity to silverbacks collectively when they had young infants compared to when they did not (with young infants: mean = 0.19, SD = 0.09; without young infants: mean = 0.16, SD = 0.07; β = 0.031, SE = 0.019, z = 1.64, p = 0.100, n = 26 dyads per female condition).

#### Change in proportion of samples in close proximity (all dyads)

**Fig 2:**
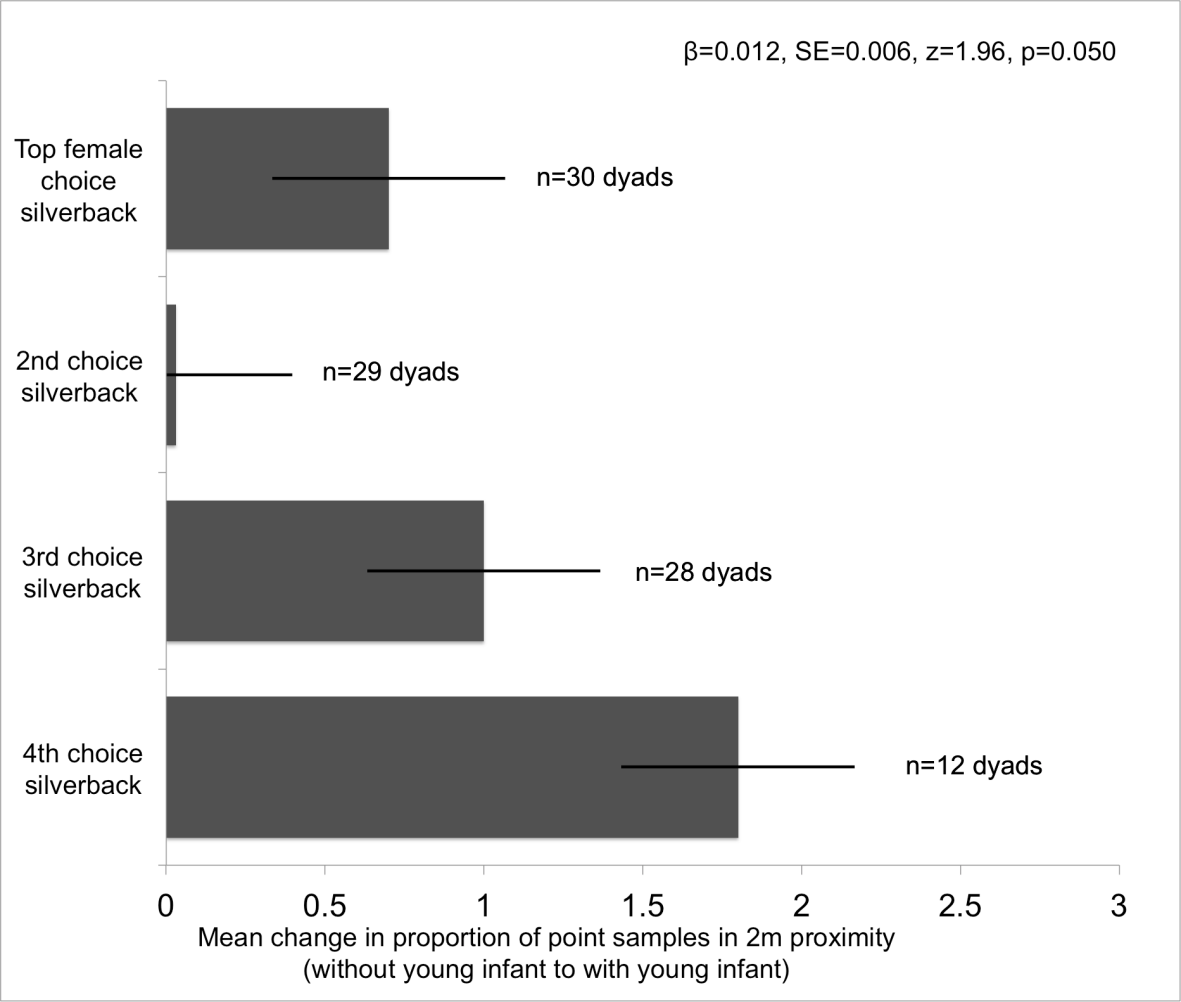
Proportionally, females in multi-male groups increased their time near males they had previously spent little time near more than they increased time near males they previously preferred (p = 0.050).

Females with young infants showed the largest increase in the proportion of point samples near silverbacks they had spent the least time near when they did not have young infants (p = 0.050; Fig 2, Table 3A). The 4^th^-choice dyads—females’ previously least-preferred males—contained an outlier whose close proximity jumped from 0% of point samples when the female did not have a young infant to 11% when she did, but with this dyad removed from the model a trend remains (Table 3B). There were insufficient approach/leave interactions to evaluate which partner was primarily responsible for this result.

**Table 3:** All male-female dyads in multi-male groups.

**3A** Mean % change, proportion of point samples in 2m proximity (Wald chi2: 4.43, p = 0.109)							
	**Coef**	**Std Err**	**Z**	**P>|z|**	**95% CI (lower)**	**95% CI (upper)**	**n (dyads)**
Female choice rank*	0.012	0.006	1.96	**0.050**	0.000	0.023	α = 30, β = 29, γ = 28, δ = 12
Silverback dominance rank (all)	-0.010	0.005	-1.91	*0*.*056*	-0.021	0.000	α = 23, β = 26, γ = 25, δ = 25
Constant	0.007	0.012	0.61	0.543	-0.016	0.030	
**3B** Mean % change, proportion of point samples in 2m proximity (outlier removed) (Wald chi2: 4.29, p = 0.117)							
Female choice rank	0.010	0.006	1.74	*0*.*082*	-0.001	0.022	α = 30, β = 29, γ = 28, δ = 11
Silverback dominance rank (all)	-0.011	0.005	-2.02	**0.044**	-0.021	-0.000	α = 23, β = 26, γ = 25, δ = 24
Constant	0.010	0.011	0.88	0.378	-0.012	0.033	

*Males were grouped by the proportion of point samples each female spent in close proximity to them in relation to all other available males, when the females did not have young infants. The male a female was in close proximity to most often was ranked a 1, second most often a 2, etc. Females with young infants increased the proportion of point samples in close proximity to silverbacks they previously were rarely close to, more than they increased the proportion of samples near previously preferred silverbacks.

### Prediction 2

In all groups, females with young infants will spend more time actively affiliating with male partners than females without young infants do.

#### Dominant silverbacks

**Fig 3:**
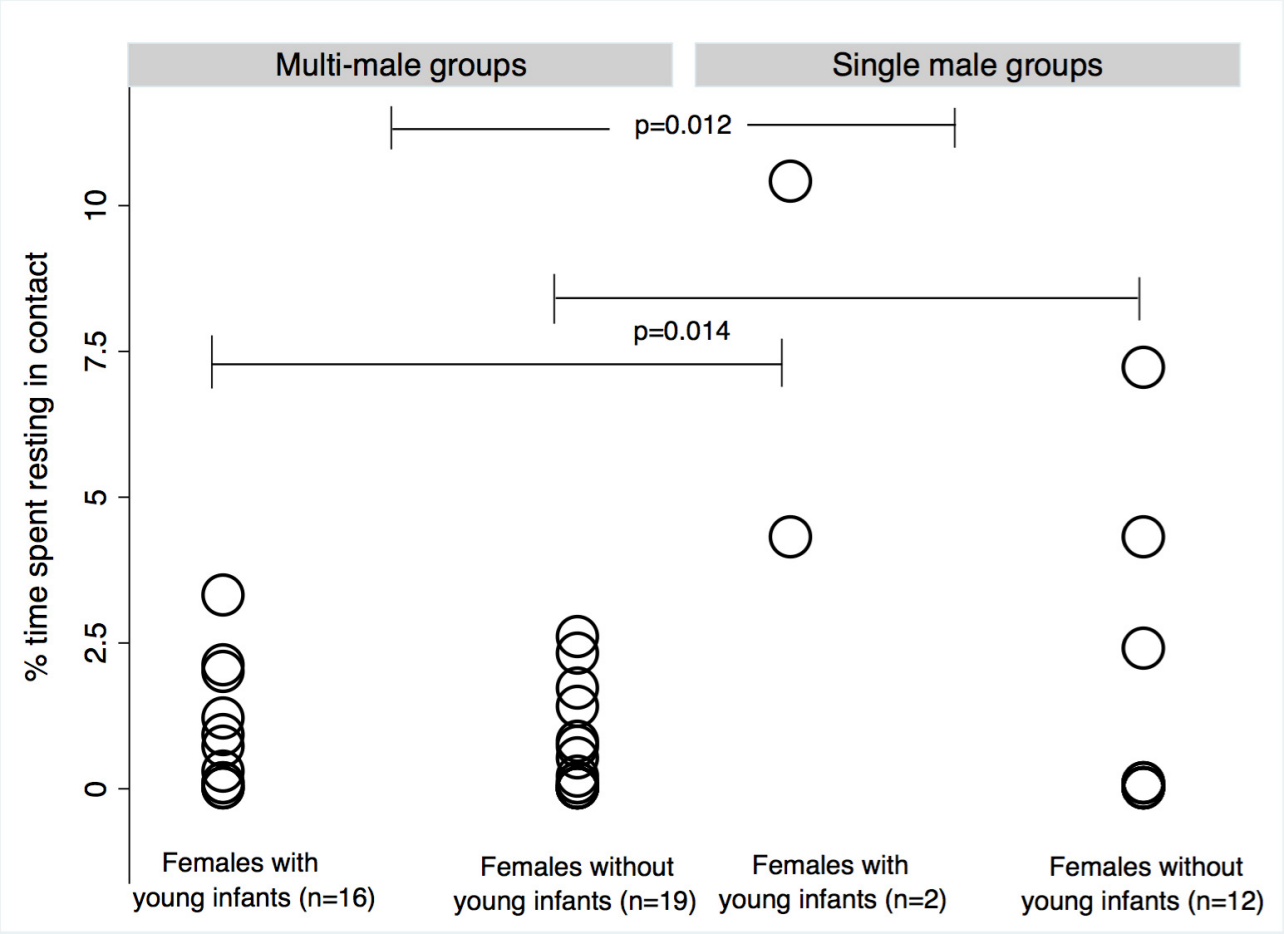
Females with young infants spent more time resting in contact with dominant males than females without young infants did. Females in single male groups appeared to drive this result, though overall rates of resting in contact were higher in single male than in multi-male groups regardless of whether females had young infants.

Females with young infants spent a significantly greater percentage of time resting in contact (mean = 1.6%, SD = 3.0%, n = 18 dyads) with dominant males than did other females (mean = 0.7%, SD = 0.9%, n = 31 dyads). This pattern is more pronounced for females with young infants in single male groups (Fig 3, Table 4A). The small available sample of females with young infants in single male groups precluded testing for interaction effects between group type and female condition for resting in contact, and also for grooming and following. However, some grooming and following values were also strikingly high among females with young infants in single male groups.

**Table 4:** Male-female dyads containing dominant silverbacks.

**4A** Percent time resting in contact (Wald chi2: 49.24, p<0.000)							
	**Coef**	**Std Err**	**Z**	**P>|z|**	**95% CI (lower)**	**95% CI (upper)**	**n (dyads)**
Female condition^t^	0.013	0.005	2.46	**0.014**	0.003	0.024	MYI^y^ = 18 WYI^ = 31
Group type^x^	-0.015	0.006	-2.50	**0.012**	-0.027	-0.003	Single male = 14 Multi-male = 35
Constant	0.016	0.005	3.20	0.001	0.006	0.025	
**4B** Percent time grooming (Wald chi2: 2.18, p = 0.337)							
Female condition	0.001	0.001	1.37	0.171	-0.001	0.003	MYI = 21 WYI = 36
Group type	-0.002	0.002	-0.72	0.472	-0.006	0.003	Single male = 13 Multi-male = 44
Constant	0.002	0.002	1.19	0.235	-0.001	0.006	
**4C** Rate at which females followed males (Wald chi2: 29.95, p<0.000)							
Female condition	0.511	0.308	1.66	*0*.*097*	-0.093	1.116	MYI = 22 WYI = 35
Group type	-1.818	0.332	-5.47	**0.000**	-2.469	-1.167	Single male = 15 Multi-male = 42
Constant	-2.815	0.211	-13.32	0.000	-3.229	-2.401	

^t^ = Comparison of females with and without young infants; reference category is females without young infants.

^x^ = Comparison of single male versus multi-male groups; reference category is single male groups.

^y^MYI = Mothers of young infants.

^WYI = Females without young infants.

These females (n = 2) groomed with their available male 3.8% and 0.1% of total focal time. In the same groups females without young infants groomed with them an average of 0.3% of focal time (SD = 0.8%, min = 0, max = 2.8%, n = 11 dyads). Females in multi-male groups groomed with dominant males an average of 0.1% of focal time when they had young infants (SD = 0.3%, min = 0, max = 1.4, n = 19 dyads), and 0.1% when they did not (SD = 0.1%, min = 0, max = 0.7, n = 25 dyads). Neither female condition nor group type were related to rates of grooming between dominant females and silverbacks (Table 4B).

In single male groups, three females with young infants followed the dominant male 0.06, 0.13, and 0.13 times/hour. For the 12 dyads in single male groups without young infants, the mean following rate was 0.07 follows/hour (SD = 0.05, min = 0, max = 0.15). In multi-male groups, following rates were significantly lower overall than in single male groups, and were identical in the two female conditions (with young infants: mean = 0.02 follows/hour, SD = 0.03, min = 0, max = 0.09, n = 19 dyads; without young infants: mean = 0.02 follows/hour, SD = 0.03, min = 0, max = 0.11, n = 23 dyads). Overall, female condition weakly predicted following rates (Table 4C). The result was clearly driven by the very high rates of following amongst the females with young infants in single male groups.

#### Non-dominant silverbacks

As was true for dyads containing dominant males in multi-male groups, there was no difference in the amount of time females with and without young infants spent resting in contact with non-dominant silverbacks (with young infants: mean = 0.20%, SD = 0.53, n = 41 dyads; without young infants: mean = 0.20%, SD = 0.80, n = 50 dyads; S1D Table). There was a statistical trend for females without young infants to groom with non-dominant males more than females with young infants did (with young infants: mean = 0.04%, SD = 0.15, n = 40 dyads; without young infants: mean = 0.22%, SD = 1.6, n = 66 dyads; S1E Table). Female condition did not predict the rate at which females followed non-dominant males (with young infants: mean = 0.01/hour, SD = 0.03, n = 43 dyads; without young infants: mean = 0.01/hour, SD = 0.04, n = 56 dyads; S1F Table). Male rank did not predict percentage of time spent either resting in contact or grooming, nor the rate at which females followed non-dominant males (S1D–S1F Table). Interaction terms for male rank and female condition had no significant effect in the models for resting in contact, grooming, or following (S1D–S1F Table).

### Prediction 3

In multi-male groups, females will be more selective in their male social partner choice when they have a young infant than when they do not.

**Fig 4:**
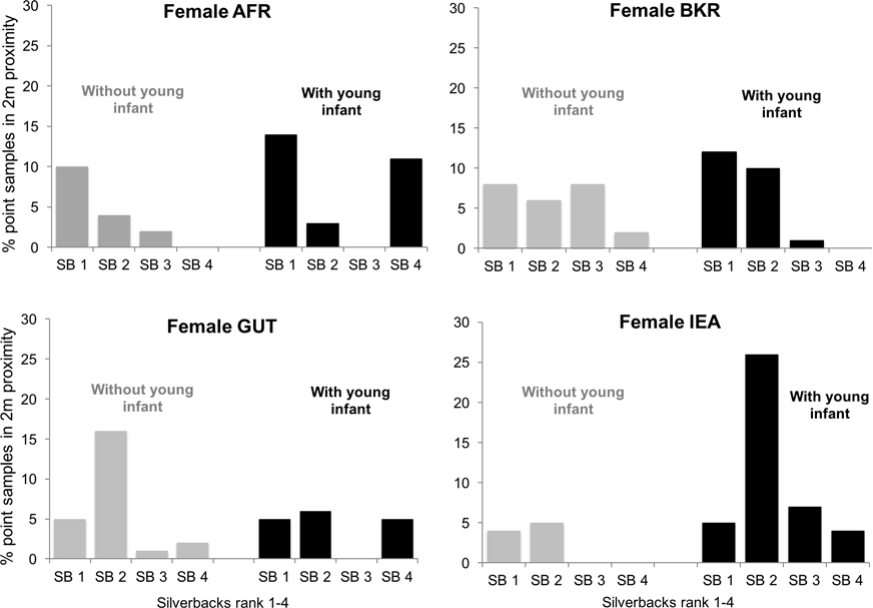
Four representative example distributions of the proportion of point samples individual females in multi-male groups spent with each available silverback when they had young infants (black bars), and when they did not (grey bars).

Females in multi-male groups demonstrated substantial variability in their association patterns with silverbacks (see examples in Fig 4). Some were frequently in close proximity to only one or two adult males, while others divided their associations more evenly. However, equitability scores were very similar in both maternal conditions (with young infants: mean = 0.194, SD = 0.100; without young infants: mean = 0.189, SD = 0.094; *t* = 0.308, p = 0.761, n = 26 dyads per condition).

### Prediction 4

At 2–3 years of age, infants in multi-male groups will prefer the same male(s) their mothers preferred in the infants’ first year of life.

We were able to evaluate this prediction for 18 infants. In 16 of 18 cases (89%), the silverback a mother was most often in close proximity to during her infant’s first year of life was also the male her infant was most often in close proximity to when the infant was two years old. In one of the 16 instances a mother had two equally preferred partners, and the infant’s most preferred partner was one of those two males (Table 5).

**Table 5:** Agreement of genetic paternity and male dominance rank with mother and infant social partner preference, operationalized as proportion of point samples spent in close proximity.

Rank of mother’s preferred male social partner when was <1 year	Rank of infant’s genetic father when infant was conceived	Rank of infant’s preferred male social partner at 2–3 years	Mother and infant prefer same male partner?	Mother most prefer infant’s genetic father?	Infant most prefer genetic father?
1	1	1	Yes	Yes	Yes
1	1	1	Yes	Yes	Yes
1	3	Infant died	N/A	No	N/A
1	2	1	Yes	No	No
1	1	1	Yes	Yes	Yes
1	1	1	Yes	Yes	Yes
1	? (NC)	1	Yes	N/A	N/A
1	1	1	Yes	Yes	Yes
1	4 (NC)	1	Yes	N/A	N/A
1	1	1	Yes	Yes	Yes
1	1	1	No	Yes	No
1 & 2 (Tied)*	3	1	Yes*	No	No
2**	3	1**	Yes	No	No
2	2	2	Yes	Yes	Yes
2	Blackback^	2	Yes	No	No
2	3	1	No	No	No
2	1	2	Yes	No	No
2	Unknown	2	Yes	Unknown	Unknown
2	1	2	Yes	No	No
			89% agreement	50% agreement	47% agreement

NC = not co-resident in infant’s social group after birth.

*Mother was in close proximity to each of the top 2 ranking silverbacks for 7% of point samples; infant was in close proximity for 8% (dominant silverback) and 0% (beta silverback) of point samples.

**Silverback was beta ranked when infant was conceived and born, dominant when infant was 2–3 years old.

^Blackbacks are males ages 8–11, and are of subordinate rank to silverbacks.

### Summary of prediction results

Females in single male groups increased the proportion of point samples spent near adult males and actively affiliated more with adult males when they had young infants compared to when they did not. Evidence for such behavioral changes in multi-male groups was much weaker; females behaved similarly toward both dominant and non-dominant males regardless of whether they had a young infant. The exceptions were 1) a statistical trend for females without young infants to groom more with non-dominant males than did females with young infants, and 2) females with young infants increased the proportion of point samples they spent in close proximity to males they previously were rarely near when they did not have young infants. Finally, females’ male social partner preferences in multi-male groups predicted their infants’ male social partner preferences when their infants were old enough to make independent choices (Table 6).

**Table 6:** Summary of females’ behavioral changes when they had young infants (<1 year old) relative to when they did not.

Did females with young infants…	Single male groups	Multi-male groups
increase close proximity to males?	Yes	No
increase active affiliation with males?	Yes*	No
distribute time amongst males differently?	N/A	No
**Did maternal social partner preference predict infant social partner preference?**	N/A	Yes

*Sample size of females with young infants in single male groups prevented statistical significance testing of group type (single male versus multi-male) and female condition (with/without young infants) interaction effects. However, in single male groups females with young infants appeared to rest in contact (Fig 3), follow, and groom with males more than either females without young infants in single male groups, or females in either condition in multi-male groups (see text under Results, Prediction 2, Dominant silverbacks).

### How do females with young infants choose silverback partners?

In all 19 cases evaluated, females’ top male social partners (measured as proportion of point samples spent in close proximity) were one of the two top-ranked males (Table 5). In half of the 16 cases in which paternity information was available and the father still resided in the group, the female’s top partner was also the infant’s father. In the remaining cases, there was a discrepancy between the identity of the mothers’ top partner and the identity of the sire.

We compared four models to determine whether male rank, paternity, both variables, or an interaction between the two best predicted the proportion of point samples females spent in close proximity to males when they had new infants (correlation coefficient = 0.32 for dominance rank and paternity; Table 5). The best-fitting model was the one that included both male rank and paternity as predictor variables (Table 7). However, the rank only and rank-paternity interaction models had low (<2) ΔAICc values. While ωAICc indicated some priority should be given to the model containing rank and paternity, there was no one model that was clearly a best fit. We therefore calculated weighted mean parameter estimates and errors for rank, paternity, and the interaction term. These clearly demonstrated that rank was the variable with the most predictive power. For both paternity and the rank-paternity interaction, standard errors were as large or larger than the beta coefficients (rank: β = -0.912, SE = 0.130; paternity: β = 0.053, SE = 0.721; rank*paternity: β = -0.366, SE = 0.415). Females were far more likely to be in close proximity to males of high rank than males of low rank when they had infants <1 year old, and paternity had no apparent predictive power.

**Table 7:** Relative fit of models predicting females’ male social partner preferences when they had young infants.

Model	LL	K	ΔAICc	ωAICc
Rank & Paternity	272.377	4	0	0.441
Rank	273.728	4	0.43	0.356
Rank*Paternity	271.984	5	1.55	0.203
Paternity	300.973	6	54.92	<0.000

LL = log likelihood; K = number of parameters estimated

ΔAICc = adjusted AIC difference values; ωAICc = adjusted

AIC weights.

## Discussion

Our prediction that females with young infants would modify their behavior toward adult males relative to periods in which they did not have young infants was partially supported. In single male groups there were meaningful increases in the proportion of time females spent in close proximity to, and actively affiliating with, the resident male in the year after infants were born. However, these changes were only observed in single male groups. Females in groups with two or more males behaved similarly toward males of all dominance ranks regardless of whether they had a young infant, with one exception: females increased the proportion of time near males they were previously rarely near relative to those they were previously frequently near. These results provide further evidence that male-female associations provide infant protection, and demonstrate that females modify their social interactions to mitigate risk to their infants. In single male groups, close relationships between males and new mothers have clear benefits for both partners. The risk of infanticide is high in single male groups, and males have high paternity certainty. In these groups, the interests of the female(s) and male are closely aligned. In multi-male groups the risk of infanticide is lower and males have less paternity certainty, which decreases the benefits of close relationships for both. We believe this best explains why females with young infants and males in single male groups were more likely to be near one another and actively affiliated more than counterpart females without young infants, or than male-female dyads in multi-male groups.

We predicted that in multi-male groups, females with young infants would concentrate their socializing on one male. Instead the opposite was true. They appeared to use a subtle form of paternity confusion after an infant was born, by increasing the proportion of time near males they spent the least time near when they did not have young infants (though note this did not result in an overall increase in close proximity to males). Although there were insufficient approach-leave interactions to directly evaluate whether the females or the males were primarily responsible for this change, in general females are more responsible for proximity maintenance outside of estrous periods than males are [58, 91]. Even if overall time together is still low, familiarizing all silverbacks to the new infant may help deter intragroup aggression or infanticide, and encourage male participation in defense should it be necessary. We speculate that too close an association with one specific partner might be disadvantageous, discouraging other males from offering tolerance and protection.

There are potential alternative explanations for the differences we observed in females’ behavior in single versus multi-male groups, although we believe protection against infanticide, plus high paternity certainty, is the most compelling. In multi-male groups, females generally face higher levels of competition for space near males [59, 92]. If space near males is indeed a resource females compete over, higher-ranking females could potentially exclude lower-ranking females from accessing preferred males when they have new infants. However, in mountain gorillas female dominance hierarchies are weaker than in many primate species [93]. They may also be unstable when group composition changes [94], which certainly occurred for some of these females as other females transferred social groups during the considerable time span covered by the data. Furthermore, not all females most preferred the dominant male, and those who preferred other males had lower levels of space competition (certainly lower than two of the three single male groups examined here). Despite this the increase in proportion of point samples in close proximity to ‘most preferred’ males was negligible. Finally, increased competition in multi-male groups is not associated with weaker relationships between the dominant male and adult females [92], and even if it were this would not necessarily preclude changes in female behavior after infant births. Females could theoretically compensate for the additional competition by e.g. increasing time near other males (which did not occur) or fighting harder for access to the dominant male.

Previous studies have proposed that mothers’ behavioral changes toward adult males might be an attempt to reduce weaning conflict by providing infants with alternative social partners [58]. Since there is no apparent reason this strategy would be more beneficial in single male groups than multi-male groups, our results do not support this interpretation. However, [95] also reported changes in relationships between adult females after the birth of infants, which suggests that additional motivations beyond infanticide avoidance contribute to maternal behavioral change. More work is needed to determine whether female-female relationships are also subject to interaction effects between reproductive state and group structure.

These results raise interesting questions about the relationship among social structure, sexual conflict, and the nature of male-female relationships. For mountain gorillas and other species with variable social structures, female and male interests may align more closely in some situations than in others. In the year after females give birth, male-female relationships appeared to reflect this variation. Females in single male groups substantially altered their behavior toward adult males, which likely reflects high infanticide risk and potential for paternal investment. In contrast, females in multi-male groups did not markedly change their behavior toward adult males. This may be because females in multi-male groups perceived males to be less likely to provide infant care, or because the need for male protection against infanticide was reduced. The results underscore the importance of evaluating the nature and strength of sexual conflict in specific contexts, rather than generalizing across species or ecologies (for relevant discussions of sexual conflict see: [96, 97, 98, 99]).

In groups in which postpartum sexual conflict occurs females should encourage infants to spend time with the highest ranking and most powerful male even if he is not the infant’s genetic father, provided the male is willing to protect the infant. Our data demonstrated that females with young infants clearly used rank, which is generally a good proxy for condition [35, 100], and not paternity to choose their social partners. They might have preferentially associated with dominant silverbacks because such males are in better condition and therefore better infant defenders, or because statistically they were the most likely fathers [63, 66]. Since male gorillas apparently do not discriminate paternity [67], dominant silverbacks should be more willing to incur defense costs than other males.

However, any male who is a possible sire might do best to err on the side of infant protection. Behavioral strategies to minimize infanticide are expected to evolve in female mammals because of the very high costs of infant loss, but such losses may also be quite costly for males. While the theoretical upper bound on a male’s fitness is quite high, most male gorillas never reproduce, and some—likely the majority of males who spend their lives in mixed sex groups—may sire very few infants who survive to reproductive age [63, 65, 66]. Any measures males can take to assure the survival of their infants are critical. Male investment can be more difficult to detect than direct care provided by mothers, because it comes in various forms and may happen at discrete but crucial points in time (e.g. humans: [101, 102]; baboons: [53, 103]). While males may not incur much cost during typical day-to-day interactions with infants (i.e., what we have previously termed “low cost parenting” in this species [61]), they may occasionally incur much larger costs if they attempt to defend infants from infanticidal males or predators. More data is needed on males’ behavioral responses during high-risk events like intergroup interactions and male takeovers.

These data provide further insight into the counter strategies adopted by females to mitigate the risk of sexually selected infanticide, a major selective pressure in many mammals. They also demonstrate the remarkable individual behavioral flexibility of animals in a species with a variable social system [62]. More work is needed to determine what other social behaviors vary with specific structural features, and to evaluate the long-term outcome of females’ social strategies in different group types.

## Supporting Information

S1 Table Model output for dyads containing non-dominant males. Model main effects and interaction terms for the outcome variables: proportion of point samples in close proximity; rate of female approaches to males; rate of females leaving males; percent time spent resting in contact; percent time spent grooming; and following rates, for dyads containing non-dominant silverbacks. Wald test values are reported for models containing at least one statistically significant or trending predictor.
(DOCX)
