# Supplementary material for: Infant Mortality Risk and Paternity Certainty Are Associated with Postnatal Maternal Behavior toward Adult Male Mountain Gorillas (Gorilla beringei beringei)
Source: PLoS One. 2016 Feb 10;11(2):e0147441. doi: 10.1371/journal.pone.0147441 (PMC4749219; doi:10.1371/journal.pone.0147441)
Supplement: S1 Table — Model main effects and interaction terms for the outcome variables: proportion of point samples in close proximity; rate of female approaches to males; rate of females leaving males; percent time spent resting in contact; percent time spent grooming; and following rates, for dyads containing non-dominant silverbacks. Wald test values are reported for models containing at least one statistically significant or trending predictor. (DOCX) [file pone.0147441.s001.docx]

**S1 Table: Model output for dyads containing non-dominant males.**

Model main effects and interaction terms for the outcome variables: proportion of point samples in close proximity; rate of female approaches to males; rate of females leaving males; percent time spent resting in contact; percent time spent grooming; and following rates, for dyads containing non-dominant silverbacks. Wald test values are reported for models containing at least one statistically significant or trending predictor.

**Table S1A-F** Male-female dyads containing non-dominant silverbacks)

|  | **Coef.** | **Std. Err.** | **Z** | **P>\|z\|** | **95% CI**  **(lower)** | **95% CI**  **(upper)** | **n**  **(dyads)** |
| --- | --- | --- | --- | --- | --- | --- | --- |
| **1A Point samples in 2m proximity (Wald chi2: 43.40, p=0.000)** | | | | | | | |
| Female condition^t^ | 0.005 | 0.183 | 0.03 | 0.978 | -0.354 | 0.364 | ^y^MYI=76  ^WYI=76 |
| Male rank^x^ | -0.890 | 0.145 | -6.15 | **0.000** | -1.173 | -0.606 | β=52, γ=50,  δ=50 |
| Fem cond x Male rank | 0.043 | 0.073 | -0.58 | 0.559 | -0.100 | 0.185 |  |
| Constant | -1.485 | 0.436 | -3.41 | 0.001 | -2.340 | -0.631 |  |
| **1B Rate at which female approached non-dominant males** | | | | | | | |
| Female condition | -1.507 | 1.306 | -1.15 | 0.249 | -4.068 | 1.053 | MYI=23  WYI=27 |
| Male rank | -0.398 | 0.581 | -0.68 | 0.493 | -1.536 | 0.740 | β=22, γ=21,  δ=7 |
| Fem cond x Male rank | 0.740 | 0.591 | 1.25 | 0.210 | -0.418 | 1.898 |  |
| Constant | -1.378 | 1.243 | -1.11 | 0.267 | -3.814 | 1.058 |  |
| **1C Rate at which females left non-dominant males** | | | | | | | |
| Female condition | 0.783 | 1.104 | 0.71 | 0.478 | -1.380 | 2.946 | MYI=23  WYI=27 |
| Male rank | 0.530 | 0.475 | 1.12 | 0.264 | -0.401 | 1.460 | β=22, γ=21,  δ=7 |
| Fem cond x Male rank | -0.217 | 0.477 | -0.46 | 0.648 | -1.151 | 0.717 |  |
| Constant | -3.304 | 1.068 | -3.09 | 0.002 | -5.397 | -1.211 |  |
| **1D Percent time resting in contact** | | | | | | | |
| Female condition | -0.005 | 0.003 | -1.54 | 0.124 | -0.011 | 0.001 | MYI=41  WYI=50 |
| Male rank | -0.005 | 0.003 | -1.52 | 0.128 | -0.012 | 0.001 | β=35, γ=34,  δ=22 |
| Fem cond x Male rank | 0.002 | 0.001 | 1.54 | 0.123 | -0.000 | 0.004 |  |
| Constant | 0.019 | 0.010 | 1.84 | 0.066 | -0.001 | 0.039 |  |
| **1E Percent time grooming (Wald chi2: 4.20, p=0.241)** | | | | | | | |
| Female condition | -0.013 | 0.007 | -1.74 | *0.082* | -0.027 | 0.002 | MYI=40  WYI=66 |
| Male rank | -0.003 | 0.003 | -0.93 | 0.351 | -0.010 | 0.003 | β=41, γ=39,  δ=26 |
| Fem cond x Male rank | 0.003 | 0.002 | 1.45 | 0.147 | -0.001 | 0.008 |  |
| Constant | 0.015 | 0.011 | 1.39 | 0.165 | -0.006 | 0.035 |  |
| **1F Rate at which females followed non-dominant males** | | | | | | | |
| Female condition | -0.844 | 2.137 | -0.39 | 0.693 | -5.033 | 3.345 | MYI=43  WYI=56 |
| Male rank | -0.379 | 0.615 | -0.62 | 0.538 | -1.584 | 0.826 | β=39, γ=27,  δ=23 |
| Fem cond x Male rank | 0.185 | 0.823 | 0.22 | 0.823 | -1.429 | 1.798 |  |
| Constant | -5.240 | 1.852 | -2.83 | 0.005 | -8.870 | -1.611 |  |

^t^=Comparison of females with and without young infants; reference category is females without young infants

^x^=Only includes males holding beta rank or lower. Counts associated with n are the number of dyads containing a male that holds the given rank.

^y^MYI=Mothers of young infants

^WYI=Females without young infants
